# Cone Beam Computed Tomography (CBCT) findings of fungal sinusitis in post COVID‐19 patient: A case report

**DOI:** 10.22088/cjim.13.0.307

**Published:** 2022

**Authors:** Farida Abesi, Mona Alimohamadi

**Affiliations:** 1 Deprtment of Oral and Maxillofacial Radiology, Dental School, Babol University of Medical Sciences, Babol, Iran; 2 Department of Oral and Maxillofacial Radiology, Dental School, Mazandaran University of Medical Sciences, Sari, Iran

**Keywords:** Cone beam computed tomography (CBCT), Corona virus, Sinusitis

## Abstract

**Background:**

Fungal infections of the paranasal sinus are increasingly recognized in both normal and immunocompromised individuals. It is necessary to distinguish invasive diseases from the non- invasive as the result and prognosis of sinus treatment different in each one. CBCT imaging could help us in this regard. In this case, we describe a fungal sinusitis according to Cone Beam Computed Tomography (CBCT) findings.

**Case presentation:**

We present a case of a 48-year-old woman with diabetes mellitus referred to our Maxillofacial Radiology Center in Babol, Iran. The patient has been discharged from the hospital recently after recovering from COVID-19 Considering the background systemic disease (diabetes) and clinical and radiological findings (extension of bone destruction), fungal sinusitis (invasive form) was listed top in the differential diagnosis list , as it is the most common condition in post-COVID-19 patients.

**Conclusion:**

CBCT images are very useful for diagnosing normal anatomy variations and sinus lesions especially bone lesions .In this case, its early diagnosis led to rapid recovery of the patient.

The 2019 coronavirus disease (COVID‐19) was first reported in December 2019 in Wuhan, China. In this disease, SARS‐CoV‐2 infection is observed in upper and lower respiratory tract structures (1, 2). Observations in patients with COVID-19 from both China (3-5) and Europe^6^ have shown sinonasal symptomatology. Rhinorrhea has been reported in individual cases; for instance, in one of the nine children with COVID-19 in a cohort study, (7) one adult in a cohort of eleven (8), and one adult in another cohort of 18 with COVID-19.^2^ Although, symptoms such as fever, cough, fatigue, shortness of breath, pain and myalgia are much more seen in lower respiratory tract. However, olfactory disorders (hyposmia or anosmia) have recently attracted attention as a noticeable symptom of COVID-19 (9, 10). However, as the COVID‐19 pandemic has spread and our data about it has grown, sinonasal pathophysiology has been playing exclusively important roles in infection, transmission, and pathognomonic symptomatology. Although our knowledge of COVID‐19 continues to rapidly evolve, there are already clinically informative insights with respect to sinonasal pathophysiology that have been uncovered in the current literature (11). Fungal sinusitis may co-exist with possible association with a preexisting disease (diabetes, lung disease) or may be created as a hospital-acquired infection (12). Uncontrolled hyperglycemia causes infection due to immune system dysfunction, including decreased T-lymphocyte and neutrophil activity, decreased secretion of inflammatory cytokines, and antibody-mediated immunity disorders, as well as angiopathy, neuropathy, glycosuria, and increased polymorphonuclear leukocyte apoptosis.

Diabetic patients have been found to have polymicrobial pathogen development (13). Evaluating anatomical variations of osteomeatal complex (OMC) can help with proper diagnosis of maxillary sinus pathologies, providing appropriate treatment and consequently reducing the relevant complications, and the results indicate the sensitivity and accuracy of low-dose radiation imaging. In the initial examination of sinusitis, bone window CT scan could be used, which is usually used due to the preference of Cone beam computed tomography (CBCT) (14).

In some post-COVID-19 patients, pain has been reported in teeth or alveolar ridge. Fungal sinusitis was seen more prevalent in patients with immunodeficiency or diabetes. This case report presents a case of fungal sinusitis with unusual palatal bone resorption following COVID-19.

## Case presentation

**Figure 1:**
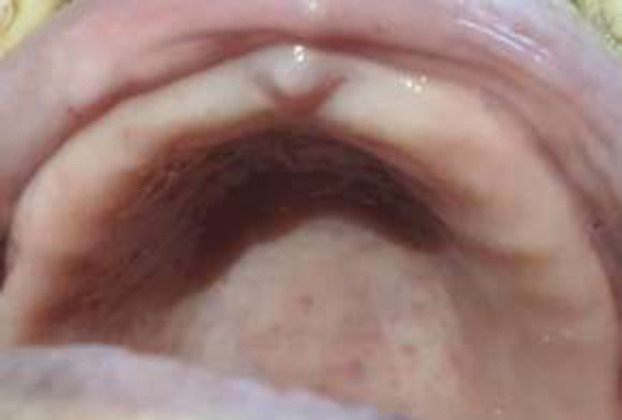
Photograph of right posterior maxillary showing normal palatal bone tissue and alveolar ridge

**Figure 2:**
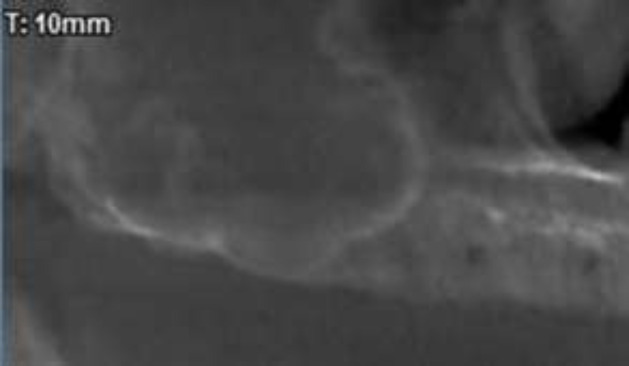
Cropped panoramic of the patient revealing no residual root and no infection but the right maxillary sinus showed increased mucosal thickening

A 48-year-old woman with diabetes mellitus referred to our Maxillofacial Radiology Center in Babol, Iran. The patient has been discharged from the hospital recently after recovering from COVID-19. She received remdesivir and steroids for the management of viral infection. On the sixth day after discharge, she visited a dentist with complaint of pain in orbital area and posterior maxillary region. In a clinical examination, the tissue of palatal bone and alveolar ridge was found normal (figure 1), but there was grayish secretion from the right nostril. The dentist ordered panoramic radiography. In panoramic view we noticed no residual root and no infection, but the right maxillary sinus showed increased mucosal thickening (figure 2).

**Figure 3:**
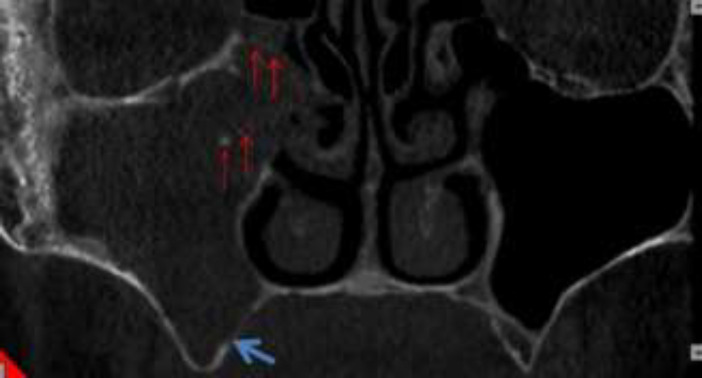
Coronal view of CBCT showing complete opacification of right maxillary sinus with calcified particles (red arrows). Notice Invasion to lateral wall of middle turbinate and the right OMC was blocked. Also, there was noticeable of palatal bone erosion (blue arrow)

**Figure 4:**
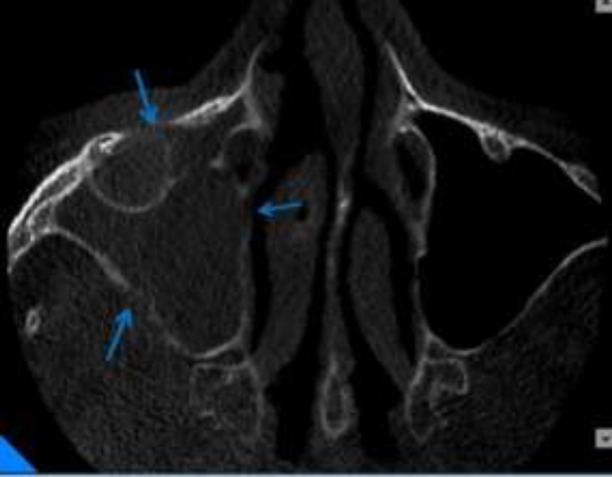
Axial view showing erosion of medial and posterolateral and anterior wall of right maxillary sinus in compared to intact and corticated left maxillary sinus walls (blue arrows)

**Figure 5:**
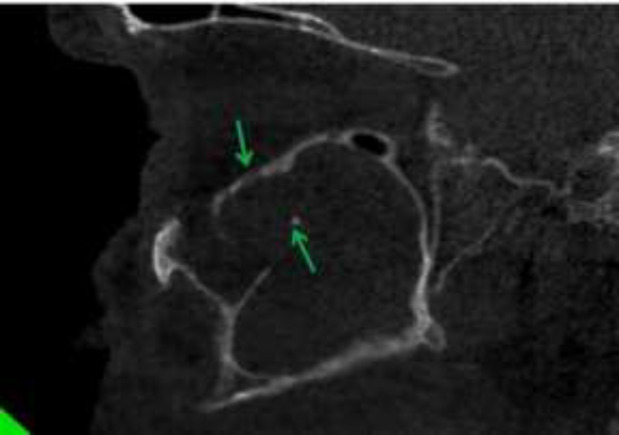
Sagittal view showing erosion of inferior border of right orbit. Also notice to calcified particle. (Green arrows)

So, we decided to take CBCT (Giano, Newtom, Verona, Italy) to observe more details of paranasal sinuses and maxillary bone. In coronal view of CBCT, complete opacification of right maxillary sinus was seen along with calcified particles near to ostium. The diameter of the biggest particle was 2 mm. Invasion to lateral wall of middle turbinate was distinct. The right OMC was blocked. Also, noticeable palatal bone erosion was observed (figure 3). In axial view, erosion of medial, posterolateral and anterior walls of right maxillary sinus was seen (figure 4). In sagittal view, erosion of inferior border of right orbit was clearly visible (figure 5).

Considering the background systemic disease (diabetes) and clinical and radiological findings (extension of bone destruction), fungal sinusitis (invasive form) was listed top in the differential diagnosis list, as it is the most common condition in post-COVID-19 patients. Chronic sinusitis was listed second. No further biopsy was taken as the patient reacted adequately to antifungal therapy.

**Figure 6:**
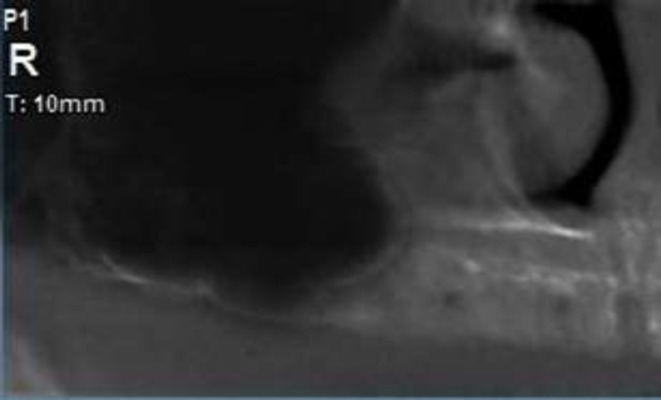
Cropped panoramic of the right maxillary sinus showing: the normal sinus without opacification

## Discussion

Fungal sinusitis is a potential infection notably found in immunocompromised patients (12). It is suggested that COVID-19 infection may cause reduction in absolute number of lymphocytes and T cells, leading to a temporary compromised immunity condition (15). To this day, we encountered a few cases of fungal sinusitis after recovering from COVID-19 diagnosed through CBCT findings. In this case, referring to dentist and taking CBCT led to the early diagnosis of fungal sinusitis.

CBCT imaging provides accurate high resolution images of anatomic structures in the maxillofacial region at lower radiation doses and costs compared to Multi Detector CT (MDCT) imaging (14, 16). There are different treatments suggested for fungal sinusitis. Invasive fungal sinusitis infection requires immediate treatment. In this patient, considering the minimal bone destruction in orbital floor, maxillary sinus walls, middle turbinate and palatal bone which could be the onset of the disease, we can be optimistic with drug treatment.

This case was also referred to ENT (ear, nose and throat) specialist. Since the patient has been discharged from the hospital recently, she did not wish to undergo a surgery for biopsy, which was one of the limitations of this article. So, the physician prescribed oral drug treatment. Patient was healed after three months. Figure 6 shows the normal sinus after the treatment period.

In conclusion, early detection of fungal sinusitis can lead to fast recovery and prevents surgery in post-COVID-19 patients.
